# Organic Catalysis for the Ring-Opening Graft Polymerization of *p*-Dioxanone with Xylan in Ionic Liquid

**DOI:** 10.3390/polym9080345

**Published:** 2017-08-06

**Authors:** Xueqin Zhang, Chuanfu Liu, Aiping Zhang, Runcang Sun

**Affiliations:** 1 State Key Laboratory of Pulp and Paper Engineering, South China University of Technology, Guangzhou 510640, China; xueqin0228@gmail.com (X.Z.); rcsun@scut.edu.cn (R.S.); 2 College of Materials and Energy, Guangdong Key Laboratory for Innovative Development and Utilization of Forest Plant Germplasm, South China Agricultural University, Guangzhou 510642, China; aiping@scau.edu.cn; 3 Beijing Key Laboratory of Lignocellulosic Chemistry, Beijing Forestry University, Beijing 10083, China

**Keywords:** xylan, poly(*p*-dioxanone), organic catalysis, ionic liquid, ring-opening graft polymerization

## Abstract

Recently, organic catalysis has become a powerful alternative to the use of more traditional metal-based catalysts. In this study, 4-dimethylaminopyridine (DMAP), 1,8-diazabicyclo[5.4.0]undec-7-ene (DBU), and 1,5,7-triazabicyclo[4.4.0]dec-5-ene (TBD) were applied to mediate the ring-opening graft polymerization (ROGP) of *p*-dioxanone (PDO) with xylan-based hemicelluloses in ionic liquid 1-butyl-3-methylimidazolium chloride ([Bmim]Cl). Excellent control of the molar ratio of the catalyst to anhydroxylose units (AXU) in xylan was found for a good tuning of the weight percent gain (WPG) of xylan-*graft*-poly(*p*-dioxanone) (xylan-*g*-PPDO) copolymers. As a result, the maximum WPG of xylan-*g*-PPDO copolymers was 431.07% (DMAP/AXU of 2/1), 316.72% (DBU/AXU of 0.2/1), and 323.15% (TBD/AXU of 0.2/1), respectively. The structure of xylan-*g*-PPDO copolymers was characterized with FT-IR and NMR. The thermal properties of copolymers were investigated using thermogravimetric analysis (TGA/DTG) and differential scanning calorimetry (DSC), and a significant difference was observed regarding the transition temperature (*T*_g_), melting temperature (*T*_m_), and crystallization temperature (*T*_c_).

## 1. Introduction

In recent years, to solve environmental issues and decrease the consumption of fossil resources, the interest in the synthesis of novel materials derived from renewable resources as the replacement of plastic materials has risen spectacularly [1,2]. Hemicelluloses, the major non-cellulose polysaccharides in wood components, are defined as heterogeneous polymers made up of xylose, mannose, arabinose, glucose, galactose, and sugar acids [3]. Generally, hemicelluloses in their natural form are non-crystalline with a low molecular weight and a degree of polymerization of 80–200 [4]. In recent years, increasing researches have been concentrated on the utilization of hemicelluloses due to their excellent biodegradability, biocompatibility, renewability, and easy availability [3,5]. Moreover, the structural varieties and diversity also make them potential biopolymers for material engineering purposes, like films [6], hydrogels [7], and additives [8]. However, hemicelluloses have the inherent drawbacks of a poor processability, poor solubility in most common organic solvents, strong hydrophilicity, and lack of thermoplasticity, limiting their further applications [9,10]. To compensate for such drawbacks, it is necessary to conduct chemical modifications on the structure of hemicelluloses [9]. Among these modifications, graft copolymerization with polymers provides an attractive and versatile way to improve the chemical and physical properties of hemicelluloses [11,12,13]. Moreover, it can combine the advantages of both natural hemicelluloses and synthetic polymers.

Among synthetic polymers, aliphatic polyesters have been the subject of a growing number of investigations. In light of the benefits that they offer, like functionality and biocompatibility, various applications or product segments are currently exhibiting high growth rates [14,15,16,17]. Previously, hemicellulosic-based copolymers had been developed by grafting with polyesters, including poly(ε-caprolactone) (PCL) [11], poly(l-lactide) (PLA) [18,19], and poly(propylene carbonate) (PPC) [20], which showed good solubility in organic solvents, a film-forming property, and thermoplasticity. Poly(*p*-dioxanone) (PPDO), which can be synthesized by the ring-opening polymerization (ROP) of *p*-dioxanone (PDO) with effective catalysts, represents the most representative and practical biopolymer [21,22]. PPDO is a semi-crystalline polymer with a melting point of about 105 °C and glass transition in the range of −15 to 8 °C [22,23,24]. Due to the excellent flexibility and bioabsorbability of PPDO, it has been applied widely as monofilament sutures, bone and tissue fixation devices, or drug delivery systems [24,25]. However, PPDO is not yet considered as a direct replacement for conventional synthetic polymers due to its high cost of production [16]. Recent advances in polymerization techniques have paved the way for a more economic production of PPDO, which can broaden its utilization [26,27,28]. The graft polymerization of PPDO onto renewable lignocellulose is an important area in the application of PPDO, as well as a practical way to alter the chemical properties of lignocellulose [29,30,31,32,33]. However, to the best of our knowledge, no research has been conducted on grafting PPDO with hemicelluloses.

Traditionally, catalysts for the ring-opening graft polymerization (ROGP) of cyclic esters with lignocellulose have been based on metallic catalysts [1,34,35]. However, metallic residues originating from these catalyst systems are difficult remove from the obtained polymers, preventing the practical applications of polymer-based materials for biomedical and electronic uses [36]. Since the first organocatalytic approach of using 4-dimethylaminopyridine (DMAP) to the ROP of lactide was reported in 2001 [37], the awareness of organic catalysts for the ROP of cyclic ester monomers has led to an increasing interest in the field of polymer synthesis [38,39]. Many organic catalysts own the advantages of simplicity, versatility, and high activity that enable them to be considered as a powerful alternative to more traditional metal-based catalysts, like stannous octoate (Sn(oct)_2_) [39,40]. In addition, organic catalysts are suitable for a range of reaction conditions, solvents, and monomers, and are typically easy to remove from the resultant polymers [39]. Generally, in the presence of alcohol initiators, the organocatalytic systems, like pyridines [41], phosphines [42,43], N-heterocyclic carbenes (NHC) [44], guanidines [2], and amidines [45] can generate linear polyesters. Of the most widely used organic catalysts are the strong bases of 1,5,7-triazabicyclo[4.4.0]dec-5-ene (TBD) and 1,8-diazabicyclo[5.4.0]undec-7-ene (DBU).

In this study, xylan-based hemicelluloses grafted PPDO copolymers (xylan-*g*-PPDO) were synthesized through ROGP with three kinds of organic catalysts: DMAP, DBU, and TBD, respectively, in ionic liquid 1-butyl-3-methylimidazolium chloride ([Bmim]Cl). The molar ratio of catalysts to anhydroxylose units (AXU) in xylan was investigated for tuning the structural characteristics and properties of xylan-*g*-PPDO copolymers. Moreover, the structural features of xylan-*g*-PPDO copolymers were studied by FT-IR, ^1^H-NMR, ^1^H-^1^H COSY, ^13^C-NMR, and ^1^H-^13^C HSQC analyses. The thermal and crystalline properties of xylan-*g*-PPDO copolymers were characterized by thermogravimetric analysis (TGA/DTG), differential scanning calorimetry (DSC), and X-ray diffraction (XRD).

## 2. Materials and Methods

### 2.1. Materials

Xylan with a xylose content of over 90% (isolated from beech wood, average molar mass of 58,000 g mol^−1^), pure PPDO (inherent viscosity of 1.5–2.2 dL/g), DMAP (purity of 99%), and DBU (purity of 99%) were purchased from Sigma-Aldrich Co., LLC (Shanghai, China). PDO with a purity of 99.5% was purchased from Xiya Reagent (Chengdu, China). TBD with a purity of 97% was supplied by Shanghai Macklin Biochemical Co., Ltd. (Shanghai, China). [Bmim]Cl with a purity of 99% was received from Cheng-Jie Chemical Co., Ltd. (Shanghai, China) and dried under vacuum for 48 h at 70 °C before use. All other chemicals were of analytical reagent grade and were used directly without further purification.

### 2.2. Synthesis of Xylan-g-PPDO Copolymers

A typical procedure to synthesize xylan-*g*-PPDO copolymers was listed as follows. Dried xylan (0.11 g, 1.67 mmol of hydroxyl groups in xylan) was suspended in 8 g [Bmim]Cl at 80 °C in a sealed 50-mL two-neck reaction flask with the protection of nitrogen. The mixture was agitated with magnetic stirring for about 1 h to obtain a xylan solution. Then, the required amount of PDO monomer (1.7 g, 16.7 mmol) and organic catalyst (as shown in Table 1) were added to the reaction flask. The ROGP reaction was allowed to proceed for about 12 h at 80 °C and was then stopped by the addition of ethanol. The mixture was further purified with ethanol to remove [Bmim]Cl, the catalyst, unreacted monomer, and byproducts, and was filtered. Following this, the obtained solid was extracted with acetone for hours and filtered until the filtrate became transparent and no precipitate appeared when a few drops of filtrate were put into ethanol, which confirmed that no byproduct remained and the purification was sufficient [46]. After that, the copolymers were dried at 50 °C under vacuum and weighed to determine the weight percent gain (WPG) of copolymers according to Equation (1). The extracted acetone was evaporated in the fume hood naturally to obtain the non-grafted PPDO homopolymer.

(1) $$\mathrm{WPG}=\frac{m2-m1}{m1}\times 100\%$$

where m1 is the weight of unmodified xylan; m2 is the weight of xylan-*g*-PPDO copolymers after the extraction in acetone.

### 2.3. Solubility Behavior

The solubility of xylan and xylan-*g*-PPDO copolymers in dimethylsulfoxide (DMSO), dimethylformamide (DMF), dichloromethane (CH_2_Cl_2_), chloroform (CHCl_3_), and acetone was investigated. A certain amount of the sample (10 mg) was added to 0.5 mL solvent and stirred at room temperature for 12 h. After the required time, the results were recorded.

### 2.4. Characterization

FT-IR spectra were measured on a Tensor 27 spectrophotometer (Bruker, Karlsruhe, Germany) from a KBr disc containing 1% (*w*/*w*) finely ground samples. Thirty-two scans were taken for each sample with a spectra width ranging from 400 to 4000 cm^−1^ at a resolution of 4 cm^−1^ in the transmittance mode.

The ^1^H-NMR, ^1^H-^1^H COSY, ^13^C-NMR, and ^1^H-^13^C HSQC spectra of unmodified xylan and xylan-*g*-PPDO copolymer were recorded from 40 mg samples in 0.5 mL of DMSO-*d*_6_ on an Avance III HD 600 M spectrometer (Bruker, Karlsruhe, Germany) with 5 mm multinuclear probe. The ^1^H-NMR and ^13^C-NMR spectra of non-grafted PPDO homopolymer were acquired in deuterochloroform (CDCl_3_) solvent.

The thermal stability (TGA/DTG) of xylan, pure PPDO, and xylan-*g*-PPDO copolymers was carried out using a Q500 thermogravimetric analyzer (TA Instruments, New Castle, DE, USA) under a nitrogen atmosphere. Thermograms were acquired between 30 °C to 600 °C at a heating rate of 15 °C/min.

DSC was carried out using a Q200 (TA Instruments, New Castle, DE, USA). The apparatus was continually flushed with nitrogen, and the flow rate was 25 mL/min. Samples (9 to 11 mg) were heated at a rate of 20 °C/min from 30 °C to 200 °C and held for 5 min. Then, the samples were cooled to −50 °C and held for 5 min. Thereafter, the samples were heated to 200 °C with the same heating rate and cooled to room temperature naturally. The transition temperature (*T*_g_), crystallization temperature (*T*_c_), and melting temperature (*T*_m_) were obtained from the thermograms. The fusion enthalpy (∆*H*_m_) values were calculated from an integration of the endothermic peaks in the DSC curves (as shown in Table 2).

XRD was determined by a D/max-III A X-ray diffractometer (Rigaku, Tokyo, Japan) in which the high-intensity monochromatic nickel-filtered Cu Kα radiation was generated at 40 kV and 40 mA. Data was collected for a diffraction angle 2θ ranging from 5° to 40° with a step size of 0.04° and a time per step of 0.2 s at room temperature.

## 3. Results

### 3.1. The Effects of Reaction Conditions on the Grafting Efficiency

**Scheme 1:**
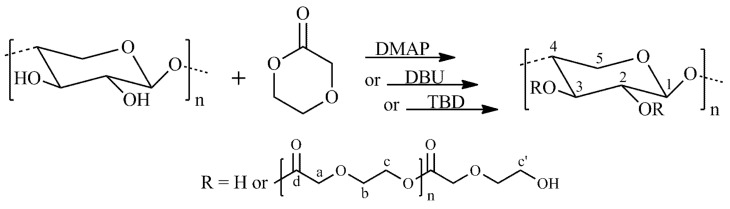
Ring-opening graft polymerization of PDO onto xylan with different organic catalysts in [Bmim]Cl.

Currently, modifying polysaccharides via ROGP is one of the most important strategies to improve their solubility, thermal processability, and compatibility. Previous studies were mainly conducted on cellulose with a metal-based catalyst like Sn(oct)_2_ [26,30]. However, the residual metals of a catalyst may affect the applications of products in biomedical materials, food packing, or electronic areas [47,48]. In this study, PPDO was polymerized by the ROGP reaction, initiated from the hydroxyl groups available on xylan and catalyzed by three kinds of organocatalysts: DMAP, DBU, and TBD in [Bmim]Cl (as shown in Scheme 1). The properties of xylan-*g*-PPDO copolymers were tuned by varying the molar ratio of catalysts to AXU, which is expressed as WPG.

As shown in Table 1, the WPG of xylan-*g*-PPDO copolymers increased from 384.51% to 431.07% when increasing the molar ratio of DMAP to AXU from 0.2:1 to 2:1. Further increasing the dosage of DMAP from 2:1 to 4:1 resulted in a decrease of WPG from 431.07% to 339.88%. Comparatively, the WPG value of xylan-*g*-PPDO copolymers mediated by DBU and TBD displayed a different trend, which was evidenced by the decrease of WPG from 316.72% to 11.09% and 323.15% to 2.55%, respectively, with the increase of the molar ratio of the catalyst to AXU from 0.2:1 to 4:1. Therefore, the optimum dosages of the catalysts in this study were 2:1 for DMAP, 0.2:1 for DBU, and 0.2:1 for TBD, respectively.

**Scheme 2:**
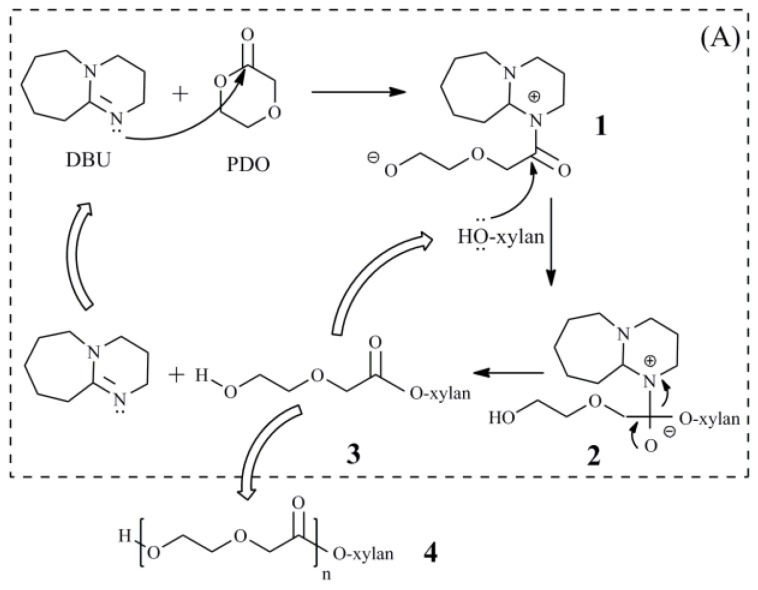

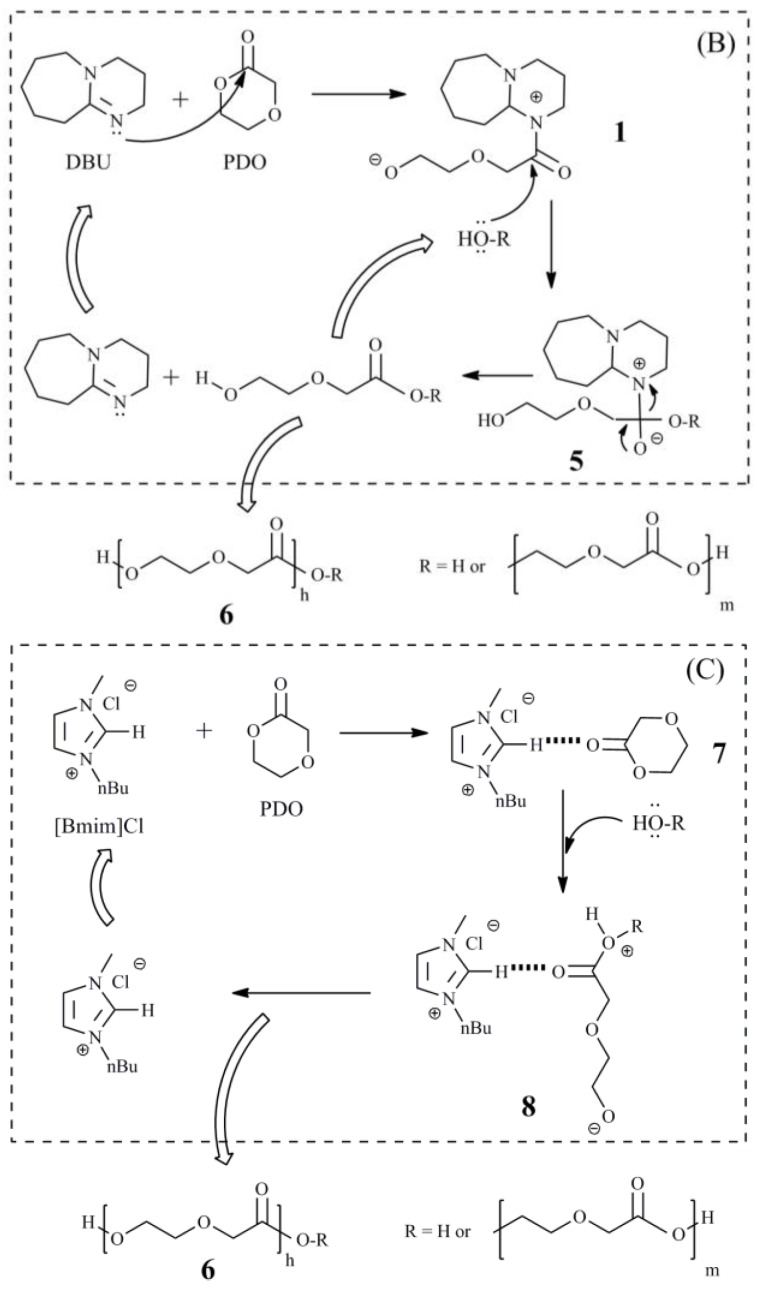
The proposed mechanisms for the ring-opening graft polymerization of PDO with xylan catalyzed by DBU (**A**) and the homopolymerization of PPDO catalyzed by DBU (**B**) or [Bmim]Cl (**C**).

**Figure 1:**
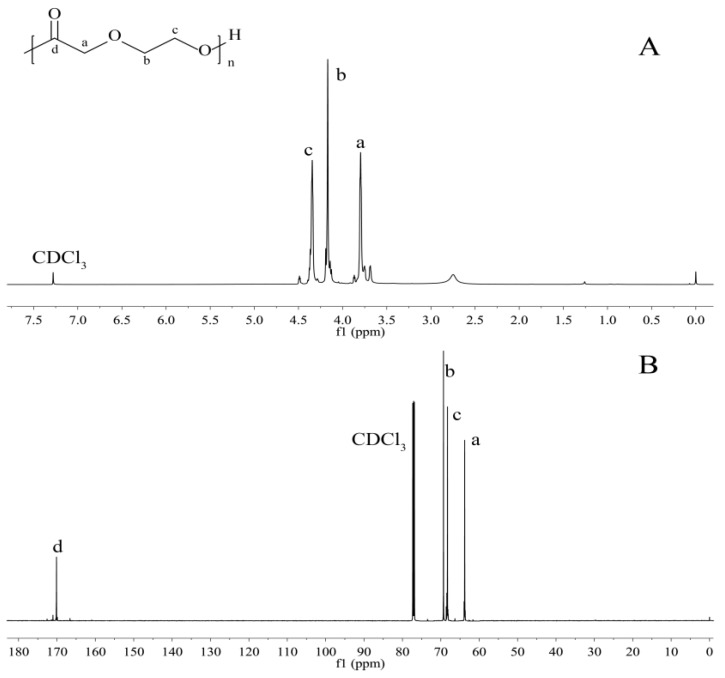
^1^H-NMR (**A**) and ^13^C-NMR (**B**) spectra of non-grafted PPDO homopolymer obtained by the extraction of xylan-*g*-PPDO sample 5 (WPG of 431.07%) in acetone.

On the basis of previous studies [30,49,50], there may be three proposed reaction mechanisms that occurred in [Bmim]Cl when utilizing organic catalysts for the ROGP of PDO with xylan (as shown in Scheme 2, using DBU as an example). In Scheme 2A, the nucleophilic attack of nitrogen in DBU to the electron-deficient carbon of the carbonyl group in PDO could generate intermediate **1** and then react with initiator hydroxyl groups in xylan to form xylan-*g*-PPDO copolymers **4**. In the present study, the excessive catalyst content could result in the decrease of the WPG of xylan-*g*-PPDO copolymers, which was mainly attributed to side-reactions, especially the homopolymerization of PPDO in ionic liquid [30,50]. As is known to all, a trace amount of water remained in the ionic liquid even after vacuum drying or freeze drying, which could act as an initiator for the homopolymerization of PPDO. In addition, the PPDO homopolymers formed were also the initiators [50]. Therefore, the possible mechanism for the homopolymerization of PPDO mediated by DBU is listed in Scheme 2B. Moreover, in light of the structural characteristic of imidazolium-based ionic liquid, the hydrogen atom at the C-2 position of [Bmim]Cl (H-2) could behave as a weak Brønsted acid catalyst by coordinating the carbonyl group of PDO to form [Bmim]Cl-PDO complex **7** (as shown in Scheme 2C) [50,51,52]. The initiation occurs when a nucleophile hydroxyl group from water or a PPDO homopolymer reacts with complex **7** and then generates the PPDO homopolymer **6** after the ring-opening chain propagation. Similar a catalytic mechanism could also be observed by DMAP or TBD catalysts. Therefore, it is necessary for the products to be post-treated with acetone to remove the PPDO homopolymers [26,30]. In the present study, the non-grafted PPDO homopolymer originating from xylan-*g*-PPDO copolymer sample 5 was obtained by the evaporation of extracted acetone in the fume hood. ^1^H-NMR and ^13^C-NMR were applied to characterize the structure of the PPDO hompopolymer (as shown in Figure 1). In ^1^H-NMR (Figure 1A), the major signals at 3.79, 4.16, and 4.24 ppm are attributed to the protons of a, b, and c of PPDO, respectively. From ^13^C-NMR (Figure 1B), the chemical shifts at 63.64, 68.26, 69.16, and 170.16 ppm are ascribed to a, c, b, and d of PPDO, respectively. These results indicated the successful removal of PPDO homopolymers from xylan-*g*-PPDO copolymers by extraction with acetone.

One of the reasons that limits the utilization of most polysaccharides is their poor solubility in organic solvents. Graft polymerization is one approach to address this problem, for the alteration in the chemical structure of the polysaccharides. In the present study, the solubility of xylan-*g*-PPDO copolymers in common organic solvents was examined, as listed in Table 1. It was found that the solubility of xylan in DMSO was developed after grafting with PPDO, probably so that the introduced PPDO side chains could disrupt the hydrogen bonds between xylan. However, in comparison to cellulose-*g*-PPDO copolymers [30], the xylan-*g*-PPDO copolymers prepared in this study could not be dissolved in DMF, probably due to the difference in the chemical structure. In addition, the xylan-*g*-PPDO copolymers were also insoluble in acetone, CH_2_Cl_2_ and CHCl_3_.

### 3.2. FT-IR Spectra

**Figure 2:**
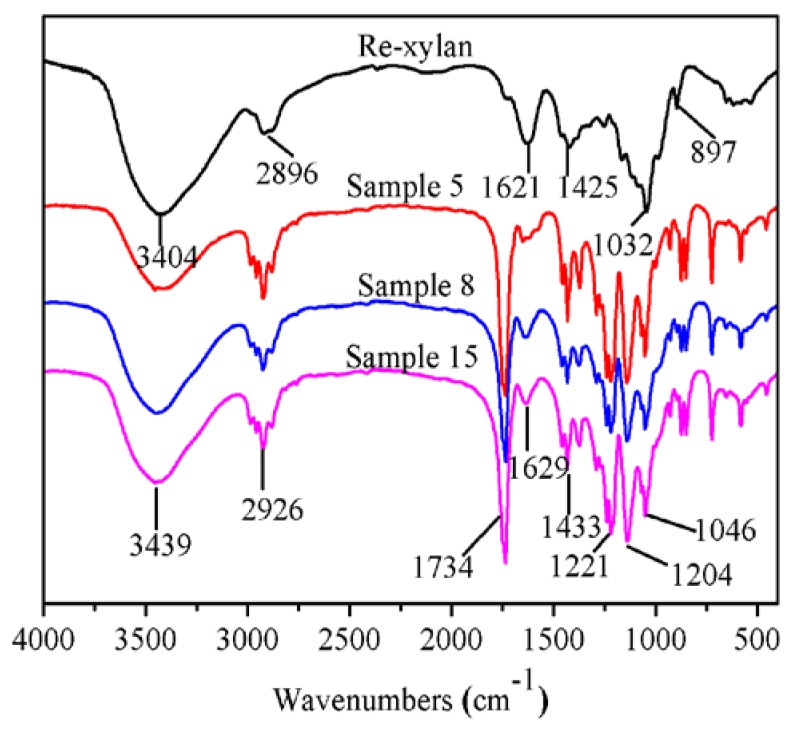
FT-IR spectra of regenerated xylan and xylan-*g*-PPDO copolymer samples 5 (WPG = 431.07%), 8 (WPG = 316.72%), and 15 (WPG = 323.15%).

FT-IR spectroscopy was applied to characterize the functional group and chemical structure of regenerated xylan and xylan-*g*-PPDO copolymer samples 5 (WPG of 431.07%), 8 (WPG of 316.72%), and 15 (WPG of 323.15%). As shown in Figure 2, the FT-IR spectrum of regenerated xylan shows a sharp band at 897 cm^−1^ characteristic of β-glucosidic linkages between the sugar units [53]. The band at 1032 cm^−1^ relates to C-O stretching in C-O-C linkages. The absorption at 1425 cm^−1^ implies the C-H and O-H bending in xylan. An intense band at 1621 cm^−1^ corresponding to the absorbed water is broader because it obscures vibrations of uronic acid carboxyl, which decreased substantially in xylan-*g*-PPDO copolymers [54]. The strong hydroxyl-stretching vibration is located at 3404 cm^−1^, and a symmetric C-H vibration band lies at 2896 cm^−1^. Comparatively, beside the characteristic absorptions of xylan, the newly appeared characteristic absorptions at 1734, 1221, and 1204 cm^−1^ for xylan-*g*-PPDO copolymer samples 5, 8, and 15 may be assigned to the stretching vibrations of C=O and C-O in PPDO side chains [55], respectively, indicating the successful ROGP of PDO with xylan. It should be noted that the O-H stretching vibration of xylan-*g*-PPDO copolymers was shifted to a higher wavenumber, at around 3439 cm^−1^, probably due to the distribution of the hydrogen bonds on the introduction of the ester group [56].

### 3.3. NMR Spectra

**Figure 3:**
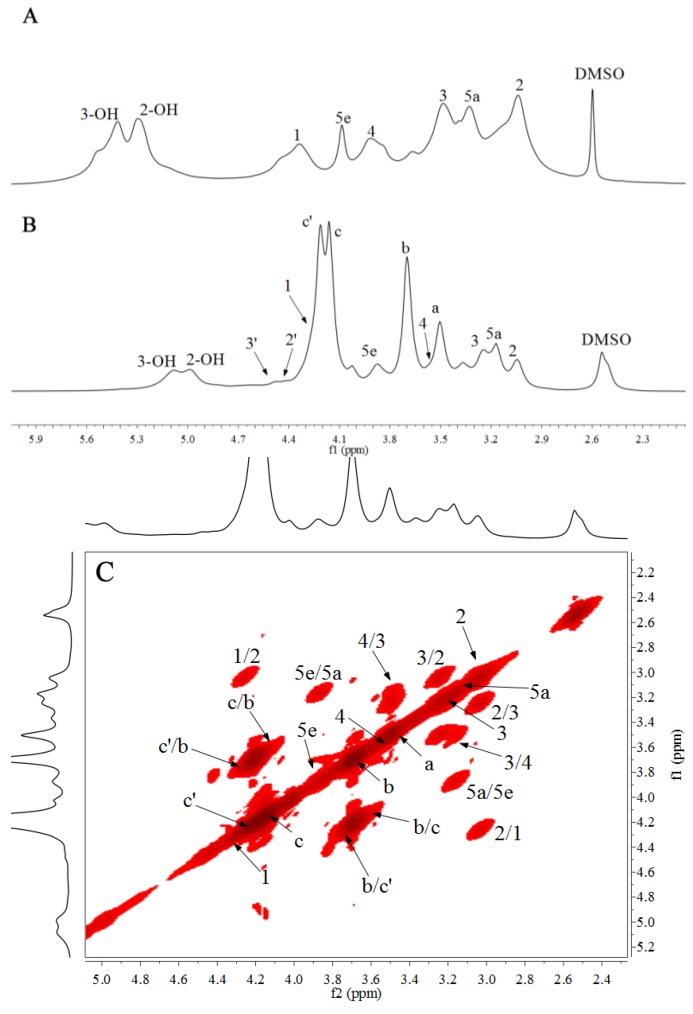

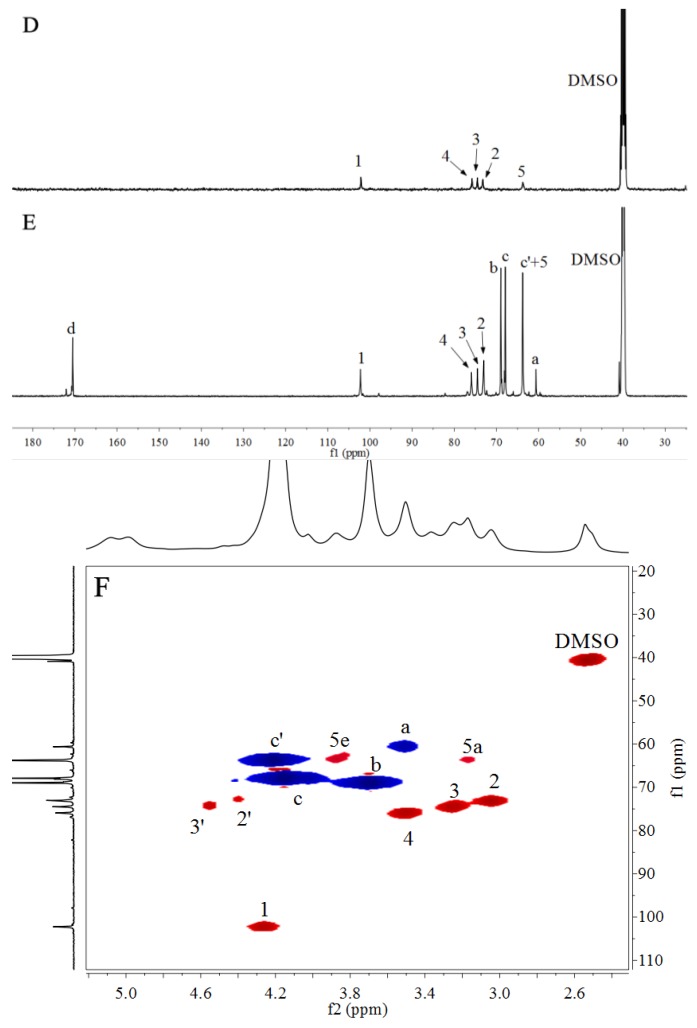
^1^H-NMR, ^1^H-^1^H COSY, ^13^C-NMR, and ^1^H-^13^C HSQC spectra of unmodified xylan (**A**,**D**) and xylan-*g*-PPDO copolymer sample 5 (**B**,**C**,**E**,**F**).

The structure of unmodified xylan and xylan-*g*-PPDO copolymer sample 5 (WPG of 431.07%) was analyzed by NMR spectroscopy (Figure 3). In Figure 3A, the major signals in the ^1^H-NMR spectrum of unmodified xylan at 3.03, 3.32, 3.48, 3.92, 4.08, and 4.34 ppm correspond to H-2, H-5a, H-3, H-4, H-5e, and H-1, respectively [57]. Moreover, the signals at 5.29 and 5.41 ppm are attributed to the protons from the hydroxyl groups in AXU [12]. Figure 3B shows the ^1^H-NMR spectrum of xylan-*g*-PPDO copolymer sample 5 (WPG of 431.07%). The signals at 3.51, 3.70, 4.17, and 4.21 ppm arise from the protons in the attached PPDO side chains of a, b, c, and c’, respectively, indicating the success of the ROGP reaction.

To confirm the correct assignment of the proton signals of the attached PPDO side chains, the ^1^H-^1^H COSY spectrum of xylan-*g*-PPDO copolymer sample 5 was investigated, as shown in Figure 3C. In order to avoid overlapping of the primary signals, the ^1^H-^1^H COSY spectrum is shown at higher contour levels to provide well-resolved correlations. The strong cross-correlations at δ_H_/δ_H_ of 3.04/3.04, 3.17/3.17, 3.24/3.24, 3.55/3.55, 3.86/3.86, 4.31/4.31, 3.04/3.26, 3.26/3.04, 3.26/3.54, 3.54/3.26, 3.17/3.86, 3.86/3.17, 3.04/4.28, and 4.28/3.04 ppm are associated with 2, 5a, 3, 4, 5e, 1, 2/3, 3/2, 3/4, 4/3, 5a/5e, 5e/5a, 2/1, and 1/2, respectively, in the xylan backbone. The cross-correlations at δ_H_/δ_H_ of 3.70/4.17, 4.17/3.70, 3.70/4.21, and 4.21/3.70 ppm are attributed to b/c, c/b, b/c’, and c’/b in the PPDO side chains, respectively, indicating the presence of a PPDO repeating unit.

Figure 3 also presents the ^13^C-NMR spectra of unmodified xylan (D) and xylan-*g*-PPDO copolymer sample 5 (E). In Figure 3D, the five major signals at 63.71, 73.24, 74.51, 75.79, and 102.13 ppm are assigned to C-5, C-2, C-3, C-4, and C-1 of the xylan backbone. In Figure 3E, the signals originating from the AXU of xylan can also be detected. In addition, the chemical shifts at 60.90, 63.77, 67.81, 69.13, and 170.37 ppm are ascribed to a, c’, c, b, and d of PPDO side chains, respectively, further indicating the successful grafting polymerization of PPDO onto xylan.

^1^H-^13^C HSQC can provide detailed information on the signal overlap in the ^1^H- and ^13^C-NMR and can be applied for qualitative and quantitative analyses of chemical structures. Therefore, the ^1^H-^13^C HSQC spectrum of xylan-*g*-PPDO sample 5 (WPG of 431.07%) is presented in Figure 3F. The primary correlations from xylan and the PPDO side chains were exhibited in a Red and Blue color, respectively. The strong correlations at δ_C_/δ_H_ of 60.44/3.50, 64.10/4.15, 67.69/4.17, and 69.10/3.70 ppm relate to C_a_/H_a_, C_c’_/H_c’_, C_c_/H_c_, and C_b_/H_b_, respectively, confirming the successful grafting of PDO with xylan. The strong correlations of C_1_/H_1_, C_2_/H_2_, C_3_/H_3_, C_4_/H_4_, C_5a_/H_5a_, and C_5e_/H_5e_ in AXU are presented at δ_C_/δ_H_ of 102.67/4.26, 73.05/3.04, 74.40/3.27, 76.40/3.52, 64.10/3.18, and 64.10/3.89 ppm, respectively. More importantly, the correlations at δ_C_/δ_H_ of 73.05/4.42 and 74.40/4.51 ppm, assigned to the substituted C_2_/H_2_ and C_3_/H_3_ (2′ and 3′, respectively), indicated the ROGP of PDO at the C_2_ and C_3_ positions of the xylan backbone. According to the integrated resonances for substituted and unsubstituted C_2_/H_2_ and C_3_/H_3_, 35.34% and 64.66% of the PPDO side chains were attached to C-2 and C-3, respectively.

### 3.4. Thermal Stability of Xylan-g-PPDO Copolymers

**Figure 4:**
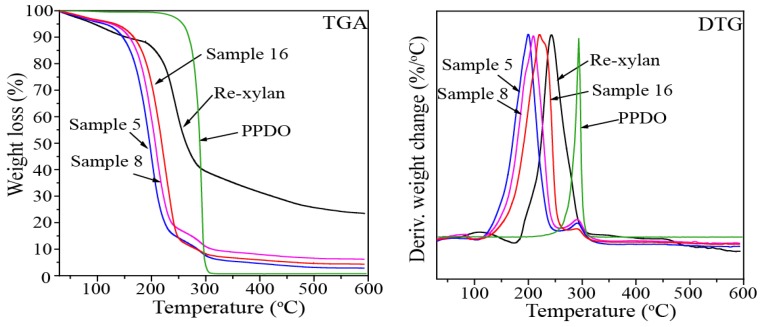
TGA and DTG curves of regenerated xylan, pure PPDO, and xylan-*g*-PPDO copolymer samples 5 (WPG = 431.07%), 8 (WPG = 316.72%), and 16 (WPG = 239.26%).

TGA/DTG analysis can provide information about the thermal decomposition profiles of derivatives, which can be used to follow the physiochemical changes that occur during modification [58]. More importantly, the thermal stability of the derivatives is an important parameter when exploring their further application. Herein, the thermal stability of regenerated xylan, xylan-*g*-PPDO copolymers, and pure PPDO was investigated by TGA/DTG (Figure 4). In the TGA curves, no apparent weight loss for pure PPDO is seen at a temperature of 100 °C, due to its inherent hydrophobic nature. A sudden drop in the curves of samples is observed during the early stage of heating. This is attributed to the loss of water, which represents about 6% and 4% of the initial weight of regenerated xylan and copolymers, respectively. In the case of xylan-*g*-PPDO copolymers, minimum weight loss was observed due to their increased hydrophobic ability after the grafting of PPDO side chains. The onset of degradation for xylan-*g*-PPDO copolymers takes place at a lower temperature than for regenerated xylan and pure PPDO, at approximately 131 °C for sample 5, 146 °C for sample 8, 160 °C for sample 16, 189 °C for regenerated xylan, and 225 °C for pure PPDO, respectively. The temperatures at 50% weight loss are about 264 °C, 290 °C, 197 °C, 207 °C, and 219 °C for regenerated xylan, pure PPDO, and samples 5, 8, and 16, respectively. These results indicated that the thermal stability of xylan decreased after the grafting of PPDO side chains. More importantly, the higher WPG of xylan-*g*-PPDO copolymers shows bigger decreases of the onset decomposition temperature. This is probably due to the introduced PPDO side chains that could break the hydrogen bonds of xylan [31]. Similar findings were obtained in chitosan-*g*-PPDO copolymers and cellulose-*g*-poly(l-lactide) (cellulose-*g*-PLLA) copolymers [31,33]. At 600 °C, the pyrolysis residues of regenerated xylan, pure PPDO, and samples 5, 8, and 16 are 24%, 0.27%, 3%, 7%, and 5%, respectively, indicating that the inorganic salts content of the samples decreased after modification [12].

In contrast with regenerated xylan, two DTG peaks of xylan-*g*-PPDO copolymers demonstrate that its degradation occurs in two steps. The first degradation peak should be caused by xylan chains, at approximately 243 °C, 221 °C, 210 °C, and 198 °C for xylan, and samples 16, 8, and 5, respectively, indicating that chemical modification led to the decreased thermal stability of xylan. For pure PPDO, the rate of thermal degradation reaches its maximum at around 294 °C. Therefore, the second degradation of xylan-*g*-PPDO copolymers occurring at around 290 °C corresponded to the grafted PPDO side chains. These results indicated that the degradation behavior of xylan-*g*-PPDO copolymers was similar to that reported in other grafted-PPDO copolymers [29,31].

### 3.5. Thermal Transition Behavior of Xylan-g-PPDO Copolymers

**Figure 5:**
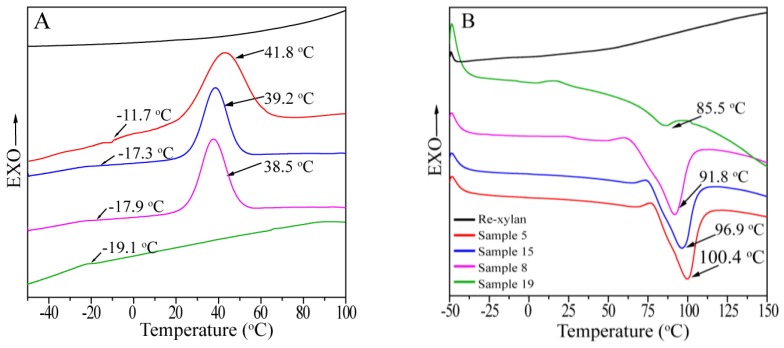
DSC cooling curves (**A**) after erasing the thermal history and subsequent heating curves (**B**) of regenerated xylan, and xylan-*g*-PPDO copolymer samples 5 (WPG = 431.07%), 8 (WPG = 316.72%), 15 (WPG = 323.15%), and 19 (WPG = 13.03%).

The samples of regenerated xylan and xylan-*g*-PPDO copolymers with various WPG were examined in thermal analysis by DSC. Figure 5A shows the cooling scans of samples after erasing the thermal history at 200 °C, and Figure 5B presents the subsequent heating curves. The data determined from DSC curves is listed in Table 2. In Figure 5A, no *T*_c_ or *T*_g_ was found for regenerated xylan. For xylan-*g*-PPDO copolymer sample 19 (WPG = 13.03%), the crystallization peak could not be detected, probably due to the shortness of PPDO side chains [29]. As the WPG of xylan-*g*-PPDO copolymers increased from 316.72% (sample 8) to 323.15% (sample 15), and 431.07% (sample 5), the temperature of crystallization increased from 38.5 °C to 39.2 °C and 41.8 °C, respectively. This is probably because the length of the PPDO side chains was long enough to allow crystallization of the copolymers during the cooling scan. These results indicated that the crystalline ability of xylan-*g*-PPDO copolymers could be influenced by its structure, as previously reported [26,29]. Moreover, as WPG increased from 13.03% to 431.07%, *T*_g_ increased from −19.1 °C to −11.7 °C, transforming to neat PPDO [23]. These results indicated that grafting with PPDO confers xylan with tunable *T*_g_, broadening its potential application as a thermoplastic [33,55]. This will be investigated in our future work.

In Figure 5B, no *T*_m_ was observed for regenerated xylan, indicating that xylan was amorphous [57]. In comparison with the DSC curve of regenerated xylan, the thermal property (*T*_m_) of xylan-*g*-PPDO copolymers was different from their substrate counterparts in the second heating scan. All of the copolymers have a *T*_m_, indicating that they are semi-crystalline. The ∆*H*_m_ determined from the DSC curves is listed in Table 2. With the increase of the WPG, both *T*_m_ and ∆*H*_m_ increased, resulting in the increased crystallinity of xylan-*g*-PPDO copolymers. This was also confirmed by the XRD results.

### 3.6. XRD Analysis

**Figure 6:**
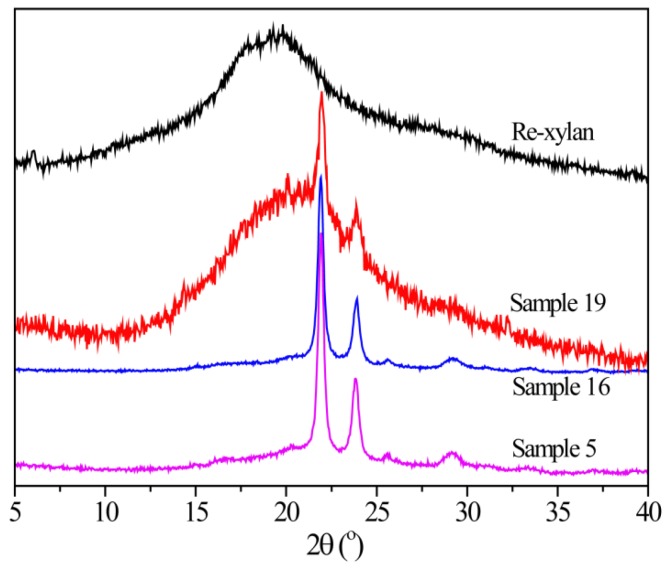
XRD patterns of regenerated xylan, and xylan-*g*-PPDO samples 5 (WPG = 431.07%), 16 (WPG = 239.26%), and 19 (WPG = 13.03%).

Structural characterization of regenerated xylan and xylan-*g*-PPDO copolymers sample 5 (WPG of 431.07%), sample 16 (WPG of 239.26%), and sample 19 (WPG of 13.03%) was determined by XRD analysis (Figure 6). From the patterns of regenerated xylan, only one disperse broad peak appeared at 2θ = 19.44°, indicating that xylan was amorphous [59]. As shown in the patterns of xylan-*g*-PPDO copolymers, three peaks at about 21.91, 23.95, and 29.20° were attributed to the crystallization of the PPDO side chain in copolymers [60]. It should be noted that sample 19 (WPG of 13.03%) still remained the main reflection of the xylan backbone, indicating that sample 19 was almost amorphous. This is probably due to the fact that the grafted PPDO was too short to form a new crystalline structure. As the WPG value increased from 13.03% to 239.26% (sample 16), and to 431.07% (sample 5), the reflection peak of xylan at about 2θ = 19.44° disappeared, presenting a similar crystalline diffraction pattern to pure PPDO [60]. This implied that the grafted PPDO was high enough to form crystallization, which was consistent with the DSC results.

## 4. Conclusions

In this study, xylan-*g*-PPDO copolymers were successfully synthesized in [Bmim]Cl with DMAP, DBU, and TBD as organic catalysts, respectively. The optimum dosages of the catalysts for the maximum WPG were 2:1, 0.2:1, and 0.2:1 for DMAP (WPG of 431.07%), DBU (WPG of 316.72%), and TBD (WPG of 323.15%), respectively, which will promote the application of these organic catalysts in the field of graft polymerization. Moreover, the *T*_g_ increased from −19.1 °C to −11.7 °C, transforming to neat PPDO, with the increase of WPG from 13.03% to 431.07%, indicating the single composition-dependent *T*_g_ of the resultant xylan-*g*-PPDO copolymers. These results suggested the transformation of xylan into thermoplastic materials with tunable *T*_g_ upon modification. Future work will be focused on the utilization of these copolymers, such as blending them with other polymers as thermoplastics or applying them as biomaterials.

## Figures and Tables

**Table 1:** Detailed structural factors of xylan-*g*-PPDO copolymers under various reaction conditions.

Sample	Catalyst	Catalyst/AXU (mol/mol)	WPG ^a^ (%)	Solubility	
				DMSO	DMF
1	DMAP	0.2:1	384.51	++	+
2	DMAP	0.5:1	394.63	++	+
3	DMAP	1:1	403.79	++	+
4	DMAP	1.5:1	403.96	++	+
5	DMAP	2:1	431.07	++	+
6	DMAP	3:1	419.98	++	+
7	DMAP	4:1	339.88	++	+
8	DBU	0.2:1	316.72	++	+
9	DBU	0.5:1	155.02	+	+
10	DBU	1:1	93.13	+	-
11	DBU	1.5:1	48.06	+	-
12	DBU	2:1	12.39	-	-
13	DBU	3:1	12.06	-	-
14	DBU	4:1	11.09	-	-
15	TBD	0.2:1	323.15	++	+
16	TBD	0.5:1	239.26	++	+
17	TBD	1:1	55.37	+	-
18	TBD	1.5:1	16.64	-	-
19	TBD	2:1	13.03	-	-
20	TBD	3:1	5.28	-	-
21	TBD	4:1	2.55	-	-

^a^ The weight percent gain of xylan due to the grafting of PPDO side chains; ++ Representing soluble; + Representing swelling; - Representing insoluble.

**Table 2:** Data obtained from DSC curves of regenerated xylan and xylan-*g*-PPDO copolymers with various WPG.

Sample	WPG (%)	*T*_c_ (°C)	*T*_g_ (°C)	*T*_m_ (°C)	∆*H*_m_ (J/g)
Re-xylan	-	-	-	-	-
PPDO ^a^	-	43.8	−9.0	107.8	58.3
5	431.07	41.8	−11.7	100.4	54.8
15	323.15	39.2	−17.3	96.9	47.2
8	316.72	38.5	−17.9	91.8	44.3
19	13.03	-	−19.1	85.5	2.48

^a^ Ref. [23].
